# Supplementary material for: Using a health belief model to assess COVID-19 vaccine intention and hesitancy in Jakarta, Indonesia
Source: PLOS Glob Public Health. 2022 Oct 28;2(10):e0000934. doi: 10.1371/journal.pgph.0000934 (PMC10021901; doi:10.1371/journal.pgph.0000934)
Supplement: Attachment — Submitted filename: Response to Reviewers.docx [file pgph.0000934.s001.docx]

Review Comments to the Author

Dear Reviewers,

Please find below our responses written in blue.

Please use the space provided to explain your answers to the questions above. You may also include additional comments for the author, including concerns about dual publication, research ethics, or publication ethics. (Please upload your review as an attachment if it exceeds 20,000 characters)

Reviewer #1: Congratulations to the research team for their thoughtful manuscript. The statistical analysis has been accurately done and presented and the findings either provides new information or consistent with other findings.

My only specific comment is the need to standardize how numbers are captured in the manuscript. For instance: the use of commas or periods in numbers/figures wasn't consistent such as IDR 10.000.001-IDR 12.500.000 (use of period in table 1 for income) or in the discussion you notice a number captured as 1,906,096 (with commas) or 11611 in table 1 (without period or commas). Same for P-values: where decimal is used (P< .05) but in the tables you see P-values reflected as 0,006 (with commas). In the discussion you have for instance n= 4,652, 40.06% (decimals used) but in the tables you see percentages using commas (not decimals for consistency). The lack of consistency is my challenge.

Recommendation: In as much as how the numbers have been reflected by the authors is not wrong, there is need for standardization and uniformity in the use of periods and commas in the tables and in the main write up to make it easier for the reader to follow. e.g either use P<0.05 or P<0,05 for consistency or 1,100,000 or 1.100.000 for consistency and not a mixture of the two formats.

Response:

Thank you for highlighting our lack of consistency; we have revised and standardized how numbers are written in the paper according to the feedback.

Reviewer #2: Thank you for inviting me to review " Using a Health Belief Model to Assess COVID-19 Vaccine Intention and Hesitancy in Jakarta, Indonesia"

Comments # 1: Abstract

Line number 43: Please add 0 (zero) before writing a decimal number and add a space between CI:2.57

Response:

Here are the revised versions:

Those with a high score of perceived susceptibility to the COVID-19 vaccine were significantly predicted vaccine hesitancy (OR = 0.18, 95% CI: 0.16 - 0.21). Perceived higher benefits of COVID-19 vaccine (OR = 2.91,. 95% CI: 2.57- 3.28), perceived severity of COVID-19 disease (OR: 1.41, 95% CI: 1.24 - 1.60), and perceived susceptibility of the current pandemic (OR= 1.21, 95% CI: 1.06 - 1.38) were significantly predicted vaccination intend.

After line 51, the authors should give a conclusion or solution-related line that beautifies the abstract part. The author can mention something like the following line. For example, the current findings on COVID-19 vaccination show that the government and policymakers should take all necessary steps to ensure the effectiveness of the vaccination program

Response:

A recommendation has been added as follows:

The current findings on COVID-19 vaccination show that the government and policymakers should take all necessary steps to remove vaccine hesitancy by increasing awareness of vaccine efficacy and benefit interventions.

Comments # 2: Introduction

Line number 87, 88: Correct the statement of only 1.178.243 persons (39.3%) received the first dose out of 3.000.689 targets. This indicates an inaccurate number, please correct the right number as 1,178,243 persons and 3,000,689 targets in place of 1.178.243 persons and 3.000.689 targets

Response:

The numbers were corrected as follows:

In addition, only 1,178,243 persons (39.3%) received the first dose out of 3,000,689 targets.

The author should include some references that have summarized vaccine acceptance and vaccine hesitancy. The author also ought to include some studies that have been conducted in developing countries like Asia and developed countries to support this manuscript

Response:

Here is the revised version according to the feedback:

In the Southeast Asia region and worldwide, studies have been conducted to examine the intention of a vaccine against COVID-19. A previous study showed that COVID-19 vaccine uptake in Indonesia was influenced by the effectiveness of the vaccines (Harapan et al., 2020). Further, COVID-19 vaccine acceptance rates in ten lower-middle income countries in Asia, Africa, and South America were higher where vaccine safety and efficacy were high (Rosiello et al., 2021). Similarly, vaccine hesitancy rates were low in Singapore (Griva et al., 2021) and New Zealand (Prickett, Habibi, & Carr, 2021).

Comments # 3: Methods

Study participants and survey design

Line number121: What is the name of the 5 districts? Please included

Response:

Quota sampling was used to analyze data collected from the proportion of gender represented across the five districts (West Jakarta, South Jakarta, East Jakarta, North Jakarta, and Central Jakarta) in the capital city, Jakarta.

Line number 125: What are the exclusion criteria of this survey? What were the reason for only adding inclusion criteria?

Response:

We have revised the sentence and included the exclusion criteria, as shown in the below sentence:

Inclusion criteria were that the respondents were Jakarta residents who were more than 18 years of age and with internet access, while those who work in Jakarta but live on the outskirts of the city were excluded from the study.

Instruments:

Line number 130: What types of questionnaires were used in this study? Was it structured, or semi-structured or other types?

Response:

A 45-item structured questionnaire was developed to assess the study objectives.

Statistical analysis

Why is not mentioned confidence interval (CI) in the statistical analysis section

Response:

We have added the CI as follows:

A P-value of less than 0.05 (95 percent of confidence interval) was considered statistically significant in this study

Comments # 4: Results

Line number 168: use space between 2.5million

Response: A space has been added: …53.68% earned less than IDR 2.5 million…

Table 1 Headings section: Please write n instead of count and % in lieu of Percentage (%)

Correct the proper percentage of table 1 in each percentage row. This column shows the inaccurate percentage and uses the full stop (.) symbol in place of a comma (,)

It is not essential to show the total number of respondents for each categorical row. It can be shown in the table 1 headline as

variable category Overall respondent N= 11611

n %

Sex Male 5844 50.33

Remove highlights in the rows of (Religion Do not answer 100)

Use single space in the row of (Have family members who are currently being or had previously diagnosed with COVID-19 No 10777 92,80). This row contains double space

Response:

Thank you for the clear guides, we have revised the table according to the feedback.

**Tabel 1** Demographic characteristics of study participants

| **Variable** | **Category N = 11611)** | **n** | **(%)** |
| --- | --- | --- | --- |
| Sex | Male | 5844 | 50.33 |
|  | Female | 5767 | 49.67 |
| Age | 18-20 years old | 170 | 1.46 |
|  | >20-30 years old | 1174 | 10.11 |
|  | >30-40 years old | 2327 | 20.04 |
|  | >40-50 years old | 3288 | 28.32 |
|  | >50-60 years old | 2347 | 20.21 |
|  | >60 years old | 2305 | 19.85 |
| Health-related jobs | Yes | 1378 | 11.87 |
|  | No | 10233 | 88.13 |
| Occupation | Student | 197 | 1.70 |
|  | Housewife | 4017 | 34.60 |
|  | Educational Staff | 275 | 2.37 |
|  | Doctor/midwife/nurse/other health workers | 99 | 0.85 |
|  | Pekerja harian tetap/tidak tetap (Ojol, pedagang, dsb) | 1136 | 9.78 |
|  | Military/Police | 59 | 0.51 |
|  | Business owner | 671 | 5.78 |
|  | State worker | 252 | 2.17 |
|  | Private worker | 2134 | 18.38 |
|  | NGO worker | 44 | 0.38 |
|  | Artist | 39 | 0.34 |
|  | Unemployed | 1256 | 10.82 |
|  | Other | 1432 | 12.33 |
| Role in local community | Youth leader | 1411 | 12.15 |
|  | Woman leader | 1690 | 14.56 |
|  | Religious leader | 328 | 2.82 |
|  | Senior citizen | 3083 | 26.55 |
|  | N/A | 5099 | 43.92 |

Table 1 continued

| **Variable** | **Category** | **n** | **%** |
| --- | --- | --- | --- |
| Monthly income (Indonesian Rupiah) | < Rp. 2.500.000 | 6233 | 53.68 |
|  | IDR 2.500.001- IDR 5.000.000 | 3697 | 31.84 |
|  | IDR 5.000.001- IDR7.500.000 | 694 | 5.98 |
|  | IDR 7.500.001-IDR 10.000.000 | 324 | 2.79 |
|  | IDR 10.000.001-IDR 12.500.000 | 139 | 1.20 |
|  | IDR. 12.500.001-IDR 15.000.000 | 116 | 1.00 |
|  | > IDR 15.000.000 | 408 | 3.51 |
| Religion | Buddha | 214 | 1.84 |
|  | Hindu | 45 | 0.39 |
|  | Islam | 10168 | 87.57 |
|  | Catholic | 468 | 4.03 |
|  | Christian | 583 | 5.02 |
|  | Indigenous Beliefs | 14 | 0.12 |
|  | Other | 19 | 0.16 |
|  | Do not answer | **100** | 0.86 |
| Education | Not finished elementary/Middle/High school | 2704 | 23.29 |
|  | High School | 6119 | 52.70 |
|  | Diploma/College/Post Graduate | 2788 | 24.01 |
| Are currently being or had previously diagnosed with COVID-19 | Yes | 612 | 5.27 |
|  | No | 10999 | 94.73 |
| Have family members who are currently being or had previously diagnosed with COVID-19 | Yes | 834 | 7.18 |
|  | No | 10777 | 92.82 |
| Comorbidities | No | 8221 | 70.80 |
|  | **Yes** | 3390 | 29.20 |
|  | Cardiovascular Disease (CVD) | 270 | 2.33 |
|  | Asthma | 290 | 2.50 |
|  | Kidney Disease | 40 | 0.34 |
|  | Diabetes Mellitus | 532 | 4.58 |
|  | Hypertension | 1355 | 11.67 |
|  | Autoimun | 31 | 0.27 |
|  | Other | 473 | 4.07 |
|  | Do not know | 833 | 7.17 |

Table 2

Similarly, it is not essential to show the total number of respondents for each categorical row. It can be written as like as table 1.

Why are not Spearman's rho and Pearson Chi-Square test analysis show different rows in a similar column? This analysis should be shown different rows in each column.

Response:

Thank you for the clear guides. We have revised the table according to the feedback, as follows:

**Table 2.** Correlation between the extended demography variables and the HBM constructs

| **Demographic variables** | | | **Perceived susceptibility of COVID-19 pandemics** | **Perceived susceptibility of COVID19 vaccine** | **Perceived severity of COVID-19 disease** | **Perceived barriers to COVID-19 vaccine** | **Perceived benefits of COVID-19 Vaccine** |
| --- | --- | --- | --- | --- | --- | --- | --- |
| Spearman's rho | Age | Correlation Coefficient | 0.022* | -0.133* | 0.004 | -0.039* | 0.046* |
|  |  | Sig. (2-tailed) | *0.016* | *0.000* | *0.638* | *0.000* | *0.000* |
|  | Monthly income (Indonesian Rupiah) | Correlation Coefficient | 0.028* | -0.120* | 0.045* | -0.137* | 0.052* |
|  |  | Sig. (2-tailed) | *0.002* | *0.000* | *0.000* | *0,000* | *0.000* |
|  | Highest Education level | Correlation Coefficient | 0.053* | -0.102* | 0094* | -0.205* | 0.03 |
|  |  | Sig. (2-tailed) | *0.000* | *0.000* | *0.000* | *0.000* | *0.007* |
| Pearson Chi-Square | Sex | Contingency Coefficient | 0.068* | 0.106* | 0.084* | 0.047* | 0.057* |
|  |  | Sig. (2-tailed) | *0.000* | *0.000* | *0.000* | *0.000* | *0.000* |
|  | Health-related jobs | Contingency Coefficient | 0.069* | 0.044* | 0.070* | 0.107* | 0.05* |
|  |  | Sig. (2-tailed) | *0.000* | *0.000* | *0.000* | *0.000* | *0.000* |
|  | Occupation | Contingency Coefficient | 0.096* | 0.146* | 0.114* | 0.139* | 0.101* |
|  |  | Sig. (2-tailed) | *0.000* | *0.000* | *0,000* | *0.000* | *0.000* |
|  | Role in Local community | Contingency Coefficient | 0.067* | 0.118* | 0.085* | 0.075* | 0.104* |
|  |  | Sig. (2-tailed) | *0.000* | *0.000* | *0.000* | *0.000* | *0.000* |
|  | Religion | Contingency Coefficient | 0.125* | 0.173* | 0.119* | 0.107* | 0.103* |
|  |  | Sig. (2-tailed) | *0.000* | *0.000* | *0.000* | *0.000* | *0.000* |

Table 2. continued

| **Demographic variables** | | | **Perceived susceptibility of COVID-19 pandemics** | **Perceived susceptibility of COVID19 vaccine** | **Perceived severity of COVID-19 disease** | **Perceived barriers to COVID-19 vaccine** | **Perceived benefits of COVID-19 Vaccine** |
| --- | --- | --- | --- | --- | --- | --- | --- |
| Pearson Chi-Square | Are currently being or had previously diagnosed with COVID-19 | Contingency Coefficient | 0.033* | 0.021 | 0.017 | 0.120* | 0.012 |
|  |  | Sig. (2-tailed) | *0.012* | *0.253* | *0.519* | *0.025* | *0.806* |
|  | Family members are currently being or had previously diagnosed with COVID-19 | Contingency Coefficient | 0.022 | 0.021 | 0.042* | 0.022 | 0.021 |
|  |  | Sig. (2-tailed) | *0.214* | *0.297* | *0000* | *0.250* | *0.288* |
|  | Comorbidities | Contingency Coefficient | 0.051* | 0,163* | 0.048* | 0.023 | 0.06* |
|  |  | Sig. (2-tailed) | *0.000* | *0.024* | *0.000* | *0.186* | *0.000* |
|  | COVID-19 Vaccination status | Contingency Coefficient | 0.037* | 0.272* | 0.073* | 0.128* | 0.139* |
|  |  | Sig. (2-tailed) | *0.003* | *0.000* | *0.000* | *0.000* | *0.000* |
|  | Family consent to get vaccinated | Contingency Coefficient | 0.065* | 0.364* | 0.075* | 0.130* | 0.282* |
|  |  | Sig. (2-tailed) | *0.000* | *0.000* | *0.000* | *0.000* | *0.000* |
|  | Vaccination willingness | Contingency Coefficient | 0.060* | 0.374* | 0.073* | 0.118* | 0.282* |
|  |  | Sig. (2-tailed) | *0.000* | *0.000* | *0.000* | *0.000* | *0.000* |

*. Correlation is significant at the 0.05 level (2-tailed).

Line number 199: Write this sentence properly. Table 2 details the relationship between whether respondents and their families were being or had previously been diagnosed with COVID-19, respondents’ comorbidities, and the HBM construct

Response:

In addition, more than half of the respondents had received vaccination when the survey was conducted (n= 7,251, 62.45%). Table 2 details the relationship between whether respondents and their families were being or had previously been diagnosed with COVID-19, respondents’ comorbidities, and the HBM construct.

Table 3:

Include coefficient symbol instead of

Use full stop (.) in place of comma (,)

Check Table 3 last rows and correct this

Line number 236: Above line number 236, insert this table in proper format and use a proper Chi-Square symbol.

Response:

We have revised the table according to the feedback, as follows:

**Table 3.** Regression analysis - predictors of the HBM construct and COVID-19 vaccine intention

| **Explanatory variable** | **ꞵ** | **Standard Error** | **P-Value** | **Wald Test** | **Odds Ratio OR** | **95% CI of OR** | |
| --- | --- | --- | --- | --- | --- | --- | --- |
|  |  |  |  |  |  | **Lower** | **Upper** |
| Perceived susceptibility to COVID-19 pandemic | 0.2 | 0.07 | 0.004 | 9.31 | 1.21 | 1.06 | 1.38 |
| Perceived susceptibility to COVID-19 vaccine | -1.71 | 0.07 | 0 | 686.5 | 0.18 | 0.16 | 0.21 |
| Perceived severity of the COVID-19 disease | 0.34 | 0.06 | 0 | 30.46 | 1.41 | 1.24 | 1.6 |
| Perceived barriers to vaccination | -0.15 | 0.06 | 0.006 | 8.78 | 0.85 | 0.77 | 0.96 |
| Perceived benefits of COVID-19 vaccine | 01.07 | 0.06 | 0 | 289.49 | 2.91 | 2.57 | 3.28 |
| Constant | 2.47 | 0.23 | 0 | 121.57 | 13 |  |  |

| **Test** | **Categories** | $\chi^{\boldsymbol{2}}$ | **DoF** | **P-Value** |
| --- | --- | --- | --- | --- |
| Overall Model Evaluation | Likelihood Ratio Test | 1686,46 | 5 | 0 |
| Goodness of Fit Test | Hosmer and Lemeshow Test | 8,48 | 8 | 0,38 |

Table 4:

Include coefficient symbol in lieu of

Use full stop (.) instead of comma (,)

Check the whole Table 4. It seems to miss information in this table.

Line number 242: Below the line number 236, insert this table in proper format and use a proper Chi-Square symbol.

Response:

Information in the table were corrected, as follows:

**Table 4.** Regression analysis - predictors of extended demography variables and COVID-19 vaccine intentions

| **Explanatory variable** | **ꞵ** | **Standard Error** | **P-Value** | **Wald Test** | **Odds Ratio OR** | **95% CI of OR** | |
| --- | --- | --- | --- | --- | --- | --- | --- |
|  |  |  |  |  |  | **Lower** | **Upper** |
| Family members who are currently or have been diagnosed with COVID-19 (Yes) | 0.40 | 0,16 | *0.01* | 6.21 | 1.50 | 1.09 | 2.05 |
| Underlying medical condition (comorbidities) (Yes) | -0.68 | 0.07 | *0.00* | 85.75 | 1.98 | 1.72 | 2.29 |
| Constant | 2.12 | 0.06 | *0.00* | 1374.20 | 8.32 |  |  |

| **Test** | **Categories** | ${\chi^{\boldsymbol{2}}}$ | **DoF** | **P-Value** |
| --- | --- | --- | --- | --- |
| Overall Model Evaluation | Likelihood Ratio Test | 87.691 | 2 | 0 |
| Goodness of Fit Test | Hosmer and Lemeshow Test | 0.19 | 1 | 0.662 |

Comments # 5: Discussions

Line number 251: Insert this reference in similar format.

Response:

The reference has been corrected into the appropriate format as follows:

By 30 April 2021, at the start of the present study, Jakarta recorded 408,620 cases, of which 6,733 died from COVID-19 (DKI Jakarta COVID-19 Statistics Bureau, 2021).

The author showed that 92.99% (10,797) would like to get vaccinated. But there is no sufficient explanation for it. The author also didn’t explain the overall world scenario of COVID-19 vaccine acceptance and hesitancy. So, the author should give some recent references to the related studies that clear the manuscript.

Response:

We have added a sentence referring to the recent findings from a relevant study as follows:

This substantial proportion of positive intention toward COVID-19 vaccination exceeds the finding that 96 countries achieved lower than the WHO target of 40% of vaccination coverage by the end of 2021 and that lower-middle-income countries could achieve between 28% to 80% vaccination coverage (Watson et al., 2022).

Line number 268: Address mathematical notation (%) in this line, we found nearly 12 percent

Response:

We have added a mathematical notation in that sentence:

While the sample in this study comprises more of the non-priority vaccine population, when we looked into the respondents’ occupations, we found nearly 12 % (n= 1,378) had healthcare-related jobs and about forty percent of the respondents (n= 4,652, 40.06%) were elderly.

In lines 269, 270, and 271, this sentence doesn’t sound right and it should be written correctly.

Response:

We have revised the sentence as follows:

Addressing vaccination barriers in Jakarta, the COVID-19 epicenter of Indonesia, is critical to ensure most of its population is protected by vaccines.

Overall comments:

There are some grammatical errors as well as improving the English language. It is noticed that the structure of many sentences is misleading. Please revise the manuscript correctly, avoiding grammatical errors.

Please add 0 (zero) before writing a decimal number

Use full stop (.) in place of comma (,) for all table components were using a numerical number

Response:

Thank you, we appreciate all feedback on the manuscript improvements.
